# Cultural adaptation and validation of the Italian version of the current relationship interview

**DOI:** 10.3389/fpsyg.2023.1250471

**Published:** 2023-09-29

**Authors:** Patrizia Velotti, Guyonne Rogier, Rosetta Castellano, Eleonora Glielmo, Valentina Alajmo, Giulio Cesare Zavattini

**Affiliations:** ^1^Department of Dynamic and Clinical Psychology, and Health Studies, Sapienza University of Rome, Rome, Italy; ^2^Department of Educational Sciences, University of Genoa, Genoa, Liguria, Italy; ^3^Institute of Specialization in Psychoanalytic Psychology of the Self and Relational Psychoanalysis, Rome, Italy; ^4^Confederation of Italian Organizations for Analytical Research on Groups (COIRAG), Rome, Lazio, Italy

**Keywords:** romantic attachment, dyadic adjustment, conflict resolution strategies, current relationship interview, adult attachment

## Abstract

**Background:**

The study of romantic relationships is based on attachment theory and the Current Relationship Interview (CRI) is a powerful tool that allows the optimal investigation of attachment representations toward romantic partners. However, evidence in this field is still unsatisfactory and further research is needed. This study aims to examine the associations between the adult attachment to partner, the style of conflict resolution, and dyadic adjustment.

**Methods:**

We administrated the Italian version of the CRI, the Dyadic Adjustment Scale (DAS), and the Rahim Organizational Conflict Inventory questionnaire - Section II (ROCI II) – to a sample of 100 heterosexual couples.

**Results:**

Individuals with preoccupied attachment reported lower levels of dyadic adjustment and men, but not women, with preoccupied attachment reported lower levels of dyadic cohesion. Levels of dyadic adjustment reported by women/men did not vary according to their attachment types. Levels of dyadic adjustment reported by couples and by women did not vary according to the matching status of attachment types between partners. However, men in romantic relationship characterized by a mismatch between attachment types reported higher levels of consensus compared to their counterparts.

**Conclusion:**

The Italian version of the CRI proves an useful tool to investigate processes underlying romantic relationships. The role of current attachment in these processes appears to be highly complex and its investigation might be impacted by methodological issues, calling for additional studies.

## Introduction

Human psychological development is strongly determined by the quality of early relationships with significant others. This is the core statement of attachment theory, elaborated by Bowlby (1962, 1980), operationalized by Ainsworth et al. (1971), and extended by Main and Goldwyn (1994). According to this perspective, the nature of the repetitive interactions between the child and their caregivers regarding the child’s attachment needs gradually shapes general representations of prototypical child-caregiver interactions in the context of attachment. These heuristics, called Internal Working Models (IWMs), typically consist of expectations that will shape the individual’s behavior in contexts triggering the activation of the attachment system. IWMs include interrelated representational components referring to the significant other, to the self, and to the relationship between the two.

The traditional tool employed to assess the nature of IWMs is the Strange Situation, a well-known observational procedure that allows to evaluate children aged 12–24 months (Ainsworth et al., 1978). In adulthood, a plethora of attachment-based instruments is available, but most are thought to assess the behavioral facet of IWMs (i.e., attachment styles) rather than their representational nature. In contrast, the Adult Attachment Interview (AAI; George et al., 1985) has been indicated as the gold standard instrument that allows to identify the nature of IWMs developed in early childhood.

### Adult attachment and romantic relationships

Despite IWMs being relatively sensitive to the changing quality of relations with caregivers in the first years of life, as the years go by, these become increasingly stable and eventually remain available throughout the entire life span. According to this framework, as a core component of personality, IWMs would impact a range of psychological functioning domains such as emotion regulation capacities or interpersonal functioning (Bowlby, 1969, 1973). A key life domain that is greatly impacted is romantic interpersonal functioning (Knies et al., 2021), with several authors stating that the quality of romantic relationships is rooted in the vicissitudes of early attachment experiences (Owens et al., 1995; Roisman et al., 2005; Velotti et al., 2014).

Although the question of the continuity of IWMs from childhood to adulthood is still under debate, a consensus has been reached towards the utility of investigating the topic of romantic relationships through the lens of the attachment theory (Hazan and Shaver, 1987; Crowell et al., 1999; Simpson and Rholes, 2012; Gray and Dunlop, 2019). Indeed, it has been stated that the attachment system would drive the individual to establish attachment bonds also with extra-familial significant others being typically friends (in adolescence) and romantic partners (in late adolescence and adulthood) (Berlin et al., 2008). The psychological functions of the old attachment figures (e.g., the parents) would be carried out by a new significant other (e.g., the romantic partner) who would be expected to satisfy the individual’s attachment needs. Again, the implicit interpersonal knowledge regarding the ways the individual’s attachment needs are framed and satisfied within this specific relationship would shape expectations towards the self, the other, and the individual’s emotional experience in the relationship. Because this specific IWM – referred to the specific romantic partner – will structure the individual’s behaviors in this current romantic bond it is expected to greatly impact on a wide range of outcomes related to this relationship.

The strong theoretical framework advancing the idea that attachment might be a key construct in the understanding of romantic relationships led several authors to develop research tools to investigate the topic. In line with the general trend in the field of research on attachment, most authors preferred the use of self-report questionnaires to assess romantic adult attachment styles. However, the Current Relationship Interview (CRI), a well-known tool to evaluate romantic IWM has been developed by Crowell and Owens (1996) to grasp the current attachment representations and stimulated the whole field of research (San Martini and Zavattini, 2011). Despite the soundness of the theoretical framework underlying the instrument and the spread of its use in several countries, it is noteworthy that no data have been published regarding the properties of the Italian version of the interview to date. An additional gap in the existing literature is related to the scarcity of data brought by the scientific community regarding the predictive value of romantic attachment, measured with the CRI, and some key outcome variables related to romantic relationship functioning. In particular, as better illustrated in the paragraph below, evidence is lacking regarding the capacity of romantic attachment – as measured by the Italian version of the CRI – to predict the perception of dyadic adjustment and styles of conflict resolution with the romantic partner.

### Romantic attachment and relationship-related outcomes

Romantic relationship quality has a relevant impact on individuals’ wellbeing. For individuals involved in an intimate relationship, such dimension assumes a central role in their life, being either a resource and/or a source of significant stress (Velotti et al., 2013; Farero et al., 2019). A plurality of terms have been used to refer to romantic relationship wellbeing including dyadic/marital adjustment (Locke and Wallace, 1959; Spanier, 1976), an umbrella term that describes the wellbeing of the relationship as an entity (Farero et al., 2019). This refers to both overall wellbeing in a relationship along with specific components (cohesion, consensus, affective expression, and satisfaction) related to the resolution of relationship difficulties (Spanier, 1976, 1979; Busby et al., 1995). The Dyadic Adjustment Scale (DAS; Spanier, 1976) is one of the most widely utilized self-report measures in clinical and research settings for measuring relationship wellbeing (Carey et al., 1993; Sabourin et al., 2005; Herrington et al., 2008; South et al., 2009). Theoretically, dyadic adjustment levels are expected to be associated with a positive, secure attachment to the partner. More precisely, these levels are expected to vary according to the impact of the individuals and their partner IWMs as well as according to the impact of the interaction between these two components (Velotti and Zavattini, 2011). Regarding this last factor, contributions highlight that beyond the quality of the attachment bond, the matching status between attachment types across the partners (i.e., being both secure, both insecure, or being mismatched), may have relevant implications for the relationship functioning (Simpson, 1990; Strauss and Morry, 2012; González-Ortega et al., 2017; Velotti et al., 2022; Cataudella et al., 2023). Empirically, few but promising pieces of research supported this perspective showing that security in attachment to partner significantly and positively predicted dyadic adjustment during the transition to parenthood (Velotti et al., 2011; Castellano et al., 2014). Of note, despite the predictive role in longitudinal studies, the cross-sectional association of security of romantic attachment as measured by the Italian version of the CRI with dyadic adjustment levels has not been tested yet.

Another central feature of romantic relationship functioning is related to the way conflicts are experienced and managed in the couple. Conflict is a natural outcome of interpersonal interactions when the parties perceive themselves as being in opposition to each other. This opposition may involve preferred outcomes, attitudes, values, and behaviors (Elsayed-Ekhouly and Buda, 1996; Chakrabarty et al., 2002). The growing interest in research on couple conflict stimulated the development of a plurality of assessment tools. For instance, the Rahim Organizational Conflict Inventory questionnaire, initially developed for and validated in organizational contexts, has been used in research on close relationships (Hammock et al., 1990; Castellano et al., 2009). Of note, IWMs may be especially influential for how partners perceive and respond to conflict (Lin, 2003; Mikulincer and Shaver, 2005; Schudlich et al., 2013). Indeed, in couples, the different points of view of each partner can lead to the onset of friction as well as explicit conflicts that affect individual and interpersonal wellbeing (Castellano et al., 2009). There may be important differences between securely and insecurely attached individuals in their approach to managing conflict within romantic relationships. Insecure attachment has been associated with relatively less adaptive or supportive conflict tactics and behaviors (Kobak and Hazan, 1991; Cohn et al., 1992; Cowan et al., 1996; Simpson et al., 1996; Guerrero et al., 2009; Sierau and Herzberg, 2012; Ricco and Sierra, 2017). However, empirical evidence regarding the link between attachment security to partner measured with the CRI and conflict resolution strategies is still lacking. Roisman et al. (2005) observed that security levels assessed with the CRI significantly discriminated between couples showing a good relationship quality in a conflict task and couples being rated with a poor relationship quality. Also, levels of coherence of the CRI transcripts resulted to be positively and significantly associated with the dyadic capacity to solve conflict assessed through an observational measure (Haydon et al., 2012). Moreover, the security of attachment to a partner measured with the CRI has been negatively correlated with the frequency of marital disagreements over the last week (Waldinger et al., 2015). An Italian study found that insecurity predicts lower levels of adaptive conflict resolution strategies during the transition to parenthood such as the cooperative integration strategy (Castellano et al., 2014).

### The present study

As briefly illustrated, despite the utility of the attachment theory in explaining romantic relationship-related processes and the uniqueness of the tool developed by Crowell and Owens (1996) – the CRI – information regarding the properties of its Italian version is lacking. In addition, the promising empirical evidence regarding the predictive role of attachment security as measured by the CRI and both dyadic adjustment and conflict resolution strategies profiles still need to be replicated and extended by additional studies. The present study aims to partially fulfil these gaps by administrating the Italian version of the CRI to a sample of adults and measuring its predictive role towards these two outcomes.

## Methods

### Participants and procedure

The study involved a total of 102 adult Italian heterosexual couples (50% males) with a mean age of 36.42 years (*S.D.* = 4.69). Regarding education levels, 39.4% of the sample report to have a middle and/or high school degree with the others having a university degree. Most couples were married (71.4%) and with children (84.1%). The group was drawn from a normal population and all participants were recruited through a convenience sampling technique. Specifically, the study was promoted thought university announcements, and students of psychology courses as well as colleagues of researchers were asked to promote the study in their networks of friends and family. Also, after the procedure was completed with a couple, participants were asked to promote the study in their personal networks.

Inclusion criteria were the following: (a) to be more than 18 years old; (b) to have a good understanding of Italian language; (c) to be involved in a romantic relationship for at least 1 year. Exclusion criteria were the following: (a) to suffer from an acute psychotic episode; (b) to have been severely intoxicated by substance or alcohol intake within the past 3 months; (c) to suffer from a neurological disease; (d) to have received a diagnosis of cognitive deficit.

Before the involvement of each participant in the research procedure, research’s aims and scopes were briefly exposed and information on privacy and anonymity was delivered. Upon reading and approval of the informed consent, participants were asked to fill several self-report questionnaires under the supervision of a psychologist. Then, a semi-structured interview was administrated and audiotaped. No compensation was given. All procedures complied with the official directions established by the American Psychological Association.

### Measures

Participants completed an initial survey (information sheet), created specially for the purpose of the study, collecting demographic information such as age, gender, educational levels and profession, marital status, and family situation.

Afterward, the nature of romantic attachment representations was investigated with the Italian version of the Current Relationship Interview (CRI; Crowell and Owens, 1996; Santona and Zavattini, 2007). The Italian version of the CRI is available upon request from the corresponding author. The purpose of the interview is to reveal how participants mentally represent attachments in romantic relationships, as reflected in their manner of speaking about their relationship. The questionnaire was built using the Adult Attachment Interview (AAI) as a model; it consists of 15 questions, the formulation of which considers the reciprocal nature of adult romantic relationships. The interview contains questions about the participant’s dating history; the nature of the present relationship and characteristics of the partner; and routine behaviors within the relationship, especially those related to providing and seeking support from the partner. To elicit an overview of the relationship, questions include topics such as what they have learned from each other and their hopes and concerns about the future of the relationship. The evaluation is based on the transcript and allows the individual to be placed in two groups: Secure (S) and Insecure depending on the profile that emerges from the scores (from 1 to 9) obtained on 18 evaluation scales. The classification system of the CRI distinguishes, within the group of individuals with insecure attachment, those who avoid talking about attachment in terms of a secure basis or who devalue it (distancing/devaluing profile - D), those who, instead, place a particular emphasis on these aspects and compulsively try to control them (preoccupied profile - P) and finally those who fall into the Unresolved category (U). Importantly, despite the administration of the interview does not require a specific training, the coding of the transcripts does. In the current study, both the administration and the coding of the transcript were performed by trained researchers.

Participants were then asked to fill two self-report questionnaires.

The way in which people tend to respond to interpersonal conflicts have been investigated using the Rahim Organizational Conflict Inventory questionnaire, Section II (ROCI II; Rahim and Majer, 1995). The questionnaire consists of 28 items on a 5-point Likert scale ranging from 1 (*Strongly Disagree*) to 5 (*Strongly Agree*) investigating the modalities of discussion with the other according to five independent responding styles: Collaborating Style, Accommodation Style, Competing Style, Avoidant Style, Compromising Style measured by 7, 6, 5, 6 and 4 statements, respectively. The internal consistencies of the scale was 0.71.

Romantic relationship quality was assessed using the Dyadic Adjustment Scale (DAS; Spanier, 1976; Gentili et al., 2002). This is a 32-item rating instrument that may be completed by either one or both partners in a relationship. Respondents are asked to rate each of the items on different Likert-type scales choosing the most suitable response options by indicating the extent of agreement or disagreement between the individual and his/her partner for each item. The most useful way of interpreting DAS is through the subscale scores: Dyadic Consensus (13 items; the degree to which the couple agrees on matters of importance to the relationship), Dyadic Satisfaction (10 items; the degree to which the couple is satisfied with their relationship), Dyadic Cohesion (5 items; the degree of closeness and shared activities experienced by the couple), and Affective Expression (4 items, the degree of demonstrations of affection and sexual relationships). A person taking this test can obtain a score from 0 to 151. The lower scores on DAS are indicative of having a problem, while the higher the score the better the person’s adjustment to the relationship. The instrument provides an estimation of the dyadic adjustment level perceived by each partner separately and by the couple (i.e., the average score obtained by each partner). In our study, the reliability of the instrument was confirmed by Cronbach’s α, with values ranging from 0.63 to 0.93.

### Statistical analyses

Cronbach’s alphas were computed to explore the internal consistency of continuous measures. Then, descriptive analyses were carried out namely frequencies, means, and standard deviations. Comparisons between groups were performed through Kruskal-Wallis tests and chi-square difference tests. All statistical analyses were performed using SPSS v.25 software for Mac.

## Results

To investigate the differences on dyadic adjustment levels across types, individuals were classified according to a four-categories (i.e., secure, insecure-dismissing, insecure-preoccupied, other) attachment model. The number of participants belonging to each group, in the whole sample and divided by gender, is displayed in Table 1. Because only one participant was classified as “Other” and no standard deviation was therefore available for this category, she was excluded from subsequent analysis.

**Table 1:** Frequencies of romantic attachment classifications in the whole sample and in the sample of men and women.

	Whole sample	Men	Women
Secure	43	16	27
Insecure dismissing	22	17	5
Insecure preoccupied	13	6	7
Other	1	1	0

### Differences in dyadic adjustment across romantic attachment classifications

Differences in dyadic adjustment levels according to romantic attachment were investigated following complementary approaches. First, we tested the hypothesis that individual perceptions of dyadic adjustment would differ according to individual romantic attachment type in the whole sample and in men and women separately. Then, we explored whether individual dyadic adjustment differed according to the partners’ romantic attachment style. Afterward, we explored whether the dyadic adjustment differed according to individuals’ romantic attachment type. Lastly, we tested the hypothesis that both individuals and dyadic estimation of adjustment would differ according to the matching between romantic attachment styles between the partners.

As displayed in Table 2, in the whole sample, individuals classified with a preoccupied attachment compared to individuals with a secure attachment obtained lower scores in the general level of dyadic adjustment. Also, men with a preoccupied attachment, compared to those with a secure attachment, obtained lower scores on the cohesion subscale of the DAS. No other significant differences were identified.

**Table 2:** Differences between individual perceptions of dyadic adjustment according attachment types.

Total sample	Secure		Dismissing		Preoccupied		*p*	*Post hoc*
	*M*	SD	*M*	SD	*M*	SD		
Adjustment	48.09	5.13	47.58	5.83	42.62	6.53	**0.039**	S>P
Cohesion	55.95	7.06	58.09	7.96	55.38	9.06	0.233	–
Affective expression	53.74	8.49	53.95	9.30	52.54	8.30	0.818	–
Satisfaction	43.05	4.57	42.18	3.42	39.38	6.89	0.089	–
Consensus	46.05	5.37	4.32	7.18	43.54	7.23	0.613	–
Men only	**Secure**		**Dismissing**		**Preoccupied**		***p***	***Post hoc***
	***M***	**SD**	***M***	**SD**	***M***	**SD**		
Adjustment	49.56	5.81	48.76	5.51	42.50	6.29	0.087	–
Cohesion	63.44	7.55	60.24	7.09	55.00	3.63	0.**041**	S>P
Affective expression	55.19	8.94	55.12	7.40	52.17	8.18	0.626	–
Satisfaction	43.44	5.33	42.88	2.80	39.83	6.55	0.143	–
Consensus	46.56	5.67	46.65	7.50	40.33	5.92	0.144	–
Women only	**Secure**		**Dismissing**		**Preoccupied**		***p***	***Post hoc***
	***M***	**SD**	***M***	**SD**	***M***	**SD**		
Adjustment	47.22	4.57	44.00	5.96	42.71	7.25	0.207	–
Cohesion	57.89	5.97	50.80	7.80	57.71	12.37	0.820	–
Affective expression	52.89	8.26	50.00	14.77	52.86	9.05	0.218	–
Satisfaction	42.81	4.14	39.80	4.55	39.00	7.66	0.953	–
Consensus	45.74	5.27	45.20	6.61	46.29	7.50	0.127	–

The bolded value indicated statistically significant result at *p* < 0.05.

Then, we investigated if women and men DAS scores significantly differed according to their attachment types. As displayed in Table 3, no significant differences were observed.

**Table 3:** Differences between women/men perceptions of dyadic adjustment according to men/women attachment types.

Women scores	Secure		Dismissing		Preoccupied		*p*	*Post-hoc*
	*M*	SD	*M*	SD	*M*	SD		
Adjustment	47.81	4.86	46.18	4.54	42.17	8.75	0.165	–
Cohesion	57.56	7.07	56.53	7.09	52.62	10.50	0.774	–
Affective Expression	55.37	8.85	51.00	9.68	52.67	7.15	0.252	–
Satisfaction	44.06	2.70	38.00	8.17	44.06	2.70	0.051	–
Consensus	47.56	4.90	46.00	5.09	42.17	8.91	0.244	–
Men scores	**Secure**		**Dismissing**		**Preoccupied**		***p***	***Post hoc***
	***M***	**SD**	***M***	**SD**	***M***	**SD**		
Adjustment	48.84	5.69	47.40	3.43	43.86	7.36	0.224	–
Cohesion	61.08	7.23	58.60	6.69	58.00	7.02	0.641	–
Affective Expression	55.88	7.52	49.20	12.09	53.14	6.72	0.274	–
Satisfaction	43.16	4.49	41.20	2.17	40.71	6.40	0.166	–
Consensus	46.32	6.59	47.40	3.58	40.29	7.80	0.144	–

Also, the hypothesis that dyadic evaluation of adjustment would differ according to attachment type was tested in the sample of women and men separately. Results, illustrated in Table 4, did not identify any significant difference.

**Table 4:** Differences between dyadic evaluations of adjustment according to men/women attachment types.

Women attachment	Secure		Dismissing		Preoccupied		*p*	*Post hoc*
	*M*	SD	*M*	SD	*M*	SD		
Adjustment	48.08	4.55	45.70	3.65	43.29	6.43	0.123	–
Cohesion	59.22	5.70	54.70	6.34	56.86	9.19	0.553	–
Affective expression	54.84	6.60	49.60	11.89	53.00	7.56	0.211	–
Satisfaction	43.02	3.78	40.50	3.14	39.86	6.93	0.510	–
Consensus	46.10	4.60	46.30	2.71	43.29	6.56	0.336	–
Men attachment	**Secure**		**Dismissing**		**Preoccupied**		***p***	***Post hoc***
	***M***	**SD**	***M***	**SD**	***M***	**SD**		
Adjustment	48.69	4.69	47.47	4.04	42.33	7.14	0.069	–
Cohesion	60.50	6.37	58.38	6.47	63.83	6.54	0.139	–
Affective expression	55.28	6.26	53.06	6.97	52.42	7.50	0.068	–
Satisfaction	43.75	3.65	42.06	6.68	38.92	7.03	0.403	–
Consensus	47.06	4.24	46.32	4.16	41.25	6.30	0.168	–

Lastly, we explored the possibility that matching status between attachment types (either matching or no matching) would have discriminant between dyadic adjustment levels. This hypothesis has been tested regarding couple levels of dyadic adjustment as well as dyadic adjustment as perceived by men and women separately. As displayed in Table 5, we found that women involved in a relationship characterized by matching attachment types between partners reported significantly lower levels of consensus compared to women involved in relationships characterized by an attachment type mismatch. To further deepen this result, we test the presence of significant differences on cohesion levels perceived by women, between couples matched on secure (*n* = 17) versus insecure (*n* = 9) attachment types. However, no significant difference emerged (*p* = 0.839).

**Table 5:** Differences between dyadic evaluations of adjustment according to matching status between attachment types.

Dyadic scores	Match (*n* = 26)		No match (*n* = 10)		*p*
	*M*	SD	*M*	SD	
Adjustment	47.00	5.27	46.90	4.91	0.915
Cohesion	58.58	6.00	57.90	8.01	0.090
Affective expression	54.58	5.80	51.90	11.42	0.620
Satisfaction	42.31	4.73	41.65	4.40	0.817
Consensus	44.83	5.09	47.90	3.60	0.764
Women scores	**Match**		**No match**		***p***
	***M***	**SD**	***M***	**SD**	
Adjustment	45.73	5.60	46.90	6.05	0.670
Cohesion	56.35	7.44	56.20	8.82	0.803
Affective expression	53.46	6.72	52.50	13.72	0.736
Satisfaction	41.54	5.50	42.30	4.88	0.482
Consensus	44.27	5.38	50.10	5.41	**0.014**
Men scores	**Match**		**No match**		***p***
	***M***	**SD**	***M***	**SD**	
Adjustment	48.27	6.03	46.90	5.93	0.435
Cohesion	60.81	6.33	59.60	8.54	0.887
Affective expression	55.69	7.08	51.30	10.66	0.168
Satisfaction	43.08	4.53	41.00	5.16	0.295
Consensus	45.38	7.39	45.70	5.68	0.631

### Differences in conflict resolution strategies across romantic attachment classifications

To test whether the distribution of constructive versus destructive conflict resolution strategies differed according to attachment type, a series of chi-square tests were performed. First, we found that conflict resolution style was not significantly more frequent among individuals with a secure, dismissing, or preoccupied attachment towards their partner. This result was replicated in the whole sample (*p* = 0.261), among men only (*p* = 0.715), and among women only (*p* = 0.115).

## Discussion

The illustrated study aimed to extend the current literature regarding current attachment in romantic relationships provided by the Italian version of the CRI.

Regarding dyadic adjustment, data towards the discriminant capacities of attachment types are lacking. First, this study suggested that preoccupied attachment may negatively impact the overall perception of dyadic adjustment. However, results failed to identify a specific dimension of dyadic adjustment accounting for this observation in the whole sample. In contrast, we found that among men, but non among women, preoccupied attachment is associated with a lack of perceived cohesion in the couple. Preoccupied attachment is known to be associated with high levels of need for approval from the significant other (Hazan and Shaver, 1987). This result may therefore suggest that men with a preoccupied attachment may be more sensitive to disagreements in the context of intimate relationships. Moreover, the fact that this result was not replicated among women, highlights the relevance of controlling for gender effect when investigating the association between attachment and processes underlying intimate relationships (Barry et al., 2015).

These findings are only partially in line with what has been found in studies carried out with self-report questionnaires, measuring romantic attachment styles that typically observed a negative association between both anxious and avoidant attachment and dyadic adjustment levels measured with the DAS (e.g., Busonera et al., 2014). This discrepancy in results supports the idea that self-report questionnaires and the CRI do measure different, albeit potentially partially overlapping, facets of the construct of romantic attachment. Also, it cannot be excluded that the estimation of the associations observed between self-report questionnaires measuring romantic attachment and the DAS may suffer from an inflation due to the similarity in the tool characteristics. In addition, the results found here partially confirm and extend what has been observed in previous longitudinal studies, using the CRI, documenting the predictive role of romantic attachment types during transitions to parenthood (Velotti et al., 2011; Castellano et al., 2014). The fact that in our cross-sectional study we found only an effect of preoccupied attachment led to two complementarily reflections. First, the role of current attachment may be especially strong in case of sensitive periods of the cycle of life that activate the attachment system (e.g., transition to parenthood) and be more hidden out of these critical time frames (i.e., in our cross-sectional study). Secondly, the exception to this rule might consist of the constant impact of preoccupied attachment on dyadic adjustment regardless of the critical nature of the life period experienced by the partners. Of note, this explanation would be in line with the idea that preoccupied attachment is characterized by a difficulty to deactivate the attachment system (Mikulincer et al., 2002).

Then, it was observed that the attachment type of an individual did not impact his/her partner’s perception of dyadic adjustment. This might indicate that romantic attachment is a factor primarily involved in the representation the individual has of his/her relationship but not, at least directly, in the perception the partner has of this relationship. Future studies conducted on larger samples or using longitudinal designs of research may further investigate this issue, for instance by testing the indirect path linking attachment type on partner perception of dyadic adjustment through an individual’s own perception of dyadic adjustment. Lastly, and in line with these findings, attachment type did not discriminate levels of dyadic adjustment perceived by the couple.

Moreover, we did not find many relevant differences in dyadic adjustment levels according to the matching status of attachment type. An exception was observed in relation to the consensus levels perceived by men, that resulted to be lower among couples with matching attachment types. This suggests that reaching agreements on relevant topics for the couple may be more important for couples with partners having divergent attachment models. Indeed, the diversity in attachment type is likely to lead to diversity in the strategies used to negotiate and solve disagreements. Therefore, couples built on a mismatch in attachment type may have a special need for finding consensus in the relationship therefore reporting higher levels of dyadic consensus on the questionnaire used.

Lastly, we tested the hypothesis that the types of conflict resolution strategies may differ across individuals with different romantic attachment models. However, we found no significant differences, suggesting that romantic attachment may not impact the way individuals negotiate conflict in intimate relationships or that other uncontrolled factors may moderate this link. Of note, our analysis was performed differentiating only between constructive and destructive conflict resolution strategies as the frequencies of some strategy types were too low to allow statistical tests. This may have provided misleading results as different attachment types may be related to specific conflict resolution strategies. Therefore, future studies with a larger sample and with a higher heterogeneity regarding prevalent conflict resolution strategies would be useful to extend our knowledge regarding this issue. Our findings are not aligned with evidence documenting an association between insecure attachment and maladaptive conflict tactics (Kobak and Hazan, 1991; Cohn et al., 1992; Cowan et al., 1996; Simpson et al., 1996; Guerrero et al., 2009; Sierau and Herzberg, 2012; Ricco and Sierra, 2017). However, these studies did not measure romantic attachment representation but attachment styles, potentially explaining the discrepancy with our data. Our observations also contrast with previous studies using the CRI and documenting an association between attachment types and variables related to conflict resolution. However, the study of Castellano et al. (2014) reported data regarding the association between romantic attachment and conflict during the transition to parenthood. Because couples recruited in this study were not necessarily under a similar stressful period eliciting or exacerbating conflicts, this may explain why we failed to grasp a significant association between attachment and conflict resolution strategies. In addition, the other studies did not exactly measure conflict resolution strategies rather than the frequency of disagreements in the last week (Waldinger et al., 2015), the capacity to solve conflict (Haydon et al., 2012), and the overall quality of relationship during a conflictual experimental situation (Roisman et al., 2005). These variables are undoubtfully related to the construct measured in our study but are not totally overlapping, evidencing the fact that conflict resolution is a highly complex topic and that results of studies investigating different facets of the construct might not be comparable.

### Limitations and future directions

Despite the value of this study, the reader should appreciate its results in light of some important limitations. First, we used a convenience sampling procedure, therefore self-selected participants may not be fully representative of the whole population of Italian couples, limiting the generalizability of our findings. This is especially true for couples seeking psychotherapy, which may imply specificities related to their clinical conditions. In addition, the small sample size limits the heterogeneity of variables investigated, reducing in turn the type of analyses that we were able to perform. For instance, only one participant was classified as having an “other” type of attachment model and this category was therefore excluded from further analyses. Also, the poor heterogeneity regarding conflict resolution strategy types did not allow for testing more complex hypotheses regarding this variable. Another issue is related to the absence of measurement of some variables that may moderate the relationships observed. For instance, emotion regulation capacities are considered to be tightly related to the attachment model and may greatly impact the outcomes measured and especially the type of conflict resolution strategies (Halperin, 2014; Garofalo et al., 2016). Lastly, the use of self-report measures to assess both dyadic adjustment and conflict resolution strategies may be considered a limitation of the study. Indeed, most of the contrasting results brought by past studies employed observational measures. This methodological issue may be further investigated in future studies to test whether the type of instrument measuring these variables may significantly impact the estimation of their link with current attachment types.

As a whole, this study highlights the complexity of the issue regarding the impact of current attachment in romantic relationships and calls for future studies investigating the topic through the CRI.

## Data availability statement

The raw data supporting the conclusions of this article will be made available by the authors, without undue reservation.

## Ethics statement

The studies involving humans were approved by Sapienza University Ethic Committee. The studies were conducted in accordance with the local legislation and institutional requirements. The participants provided their written informed consent to participate in this study.

## Author contributions

PV, RC, and GZ contributed to conception and design of the study. GR organized the database and performed the statistical analysis. EG and VA wrote the first draft of the manuscript. All authors contributed to manuscript revision, read, and approved the submitted version.
